# Processing of Continuously Provided Punishment and Reward in Children with ADHD and the Modulating Effects of Stimulant Medication: An ERP Study

**DOI:** 10.1371/journal.pone.0059240

**Published:** 2013-03-21

**Authors:** Yvonne Groen, Oliver Tucha, Albertus A. Wijers, Monika Althaus

**Affiliations:** 1 Department of Clinical and Developmental Neuropsychology, University of Groningen, Groningen, The Netherlands; 2 Department of Experimental Psychology, University of Groningen, Groningen, The Netherlands; 3 Department of Child and Adolescent Psychiatry, University Medical Center Groningen, University of Groningen, Groningen, The Netherlands; University of Western Brittany, France

## Abstract

**Objectives:**

Current models of ADHD suggest abnormal reward and punishment sensitivity, but the exact mechanisms are unclear. This study aims to investigate effects of continuous reward and punishment on the processing of performance feedback in children with ADHD and the modulating effects of stimulant medication.

**Methods:**

15 Methylphenidate (Mph)-treated and 15 Mph-free children of the ADHD-combined type and 17 control children performed a selective attention task with three feedback conditions: *no-feedback, gain and loss*. Event Related Potentials (ERPs) time-locked to feedback and errors were computed.

**Results:**

All groups performed more accurately with gain and loss than without feedback. Feedback-related ERPs demonstrated no group differences in the feedback P2, but an enhanced late positive potential (LPP) to feedback stimuli (both gains and losses) for Mph-free children with ADHD compared to controls. Feedback-related ERPs in Mph-treated children with ADHD were similar to controls. Correlational analyses in the ADHD groups revealed that the severity of inattention problems correlated negatively with the feedback P2 amplitude and positively with the LPP to losses and omitted gains.

**Conclusions:**

The early selective attention for rewarding and punishing feedback was relatively intact in children with ADHD, but the late feedback processing was deviant (increased feedback LPP). This may explain the often observed positive effects of *continuous* reinforcement on performance and behaviour in children with ADHD. However, these group findings cannot be generalised to all individuals with the ADHD, because the feedback-related ERPs were associated with the severity of the inattention problems. Children with ADHD-combined type with more inattention problems showed both deviant early attentional selection of feedback stimuli, and deviant late processing of non-reward and punishment.

## Introduction

### Objective

Attention Deficit Hyperactivity Disorder (ADHD) is a highly prevalent developmental disorder characterised by developmentally inappropriate inattentiveness, impulsivity and hyperactivity [1]. ADHD has been associated with both executive functioning and motivational deficits, including a diminished capacity to monitor behaviour and feedback [2]–[7]. Studies investigating behavioural performance on cognitive tasks have provided evidence for an abnormal sensitivity to motivational cues, e.g. reward/reinforcement and punishment, in children with ADHD, but the nature of this abnormal sensitivity remains unclear [8]. Luman and colleagues [9] concluded in their literature review on the impact of reinforcement in ADHD, that children with ADHD have problems in keeping up optimal performance when they have to rely solely on their intrinsic motivation, i.e. without external motivators such as feedback or reward [3], [6]. Across studies, reinforcement of relatively high intensity and/or immediacy was found to have a positive effect on task performance and self-reported motivation in children with ADHD [8]. There was some evidence that this positive effect on task performance was even more prominent in these children compared to typically developing (TD) children.

Motivational models of ADHD formulated several different predictions about reinforcement sensitivity [9]. For instance, models predict that individuals with ADHD have (1) a preference for small immediate reward over large delayed reward [5], [7], [10], (2) reduced neurobiological sensitivity to reward [5], [11] and reduced reward anticipation [7], [10], and (3) reduced behavioural sensitivity to cues of aversive stimuli in general [12], [13], though others predicted increased sensitivity to punishment [14], [15]. A neurocomputational model of fronto-striatal dopamine and noradrenaline function predicts that individuals with ADHD have specific deficits in learning from reinforcement (Go and NoGo learning) [11], [16]. Interestingly, some models presume that the monitoring of continuously provided external feedback is relatively intact in individuals with ADHD [10], [17]. However, the consequences of feedback might not be used to update the reinforcement history, i.e. the identification and use of reward predictors [10] or might not be implemented in the energetic state regulation to optimize behavioural performance [17]. The main aim of the present study was to gain insight into the monitoring of continuous performance feedback (i.e. immediate as well as consequent) signalling reward and punishment in children with ADHD and the modulating effects of stimulant medication (Methylphenidate, Mph). To this end, Event Related Potentials (ERPs) from the Electroencephalogram were used to identify different component processes of feedback processing; 1) the early attentional selection and detection of feedback and 2) the late processing of the affective value of feedback. The feedback was coupled with monetary gains and losses to provide insight into reward and punishment sensitivity respectively.

### Performance Feedback Sensitivity in ADHD

Only few psychophysiological studies on feedback monitoring in ADHD focussed on performance feedback that is contingent to the response, i.e. feedback that is coupled to the true performance of the participant. Making use of a feedback-based learning paradigm with performance feedback, our group previously demonstrated that children with ADHD showed a normal feedback-related frontocentral P2 amplitude, but a trend towards a reduced Late Positive Potential (LPP) in response to negative feedback stimuli [18]. The frontocentral P2 (also called P200 or Frontal Selection Positivity) has previously been associated with early selective attention and may reflect attention-facilitation by salient (target) stimuli [19]–[22]. The LPP has repeatedly been described to reflect increased sustained attention to affective-motivational stimuli [23]–[25], and its amplitude has consistently been demonstrated to be enhanced by emotionally salient events such as pleasant and unpleasant photographs, emotional faces and reward [26]. The LPP amplitude depends on how the emotional stimuli are appraised and attended to, with smaller LPP amplitudes in conditions in which the emotional stimulus is reappraised and/or is judged on its non-emotional content [26]. In the context of feedback processing the LPP may reflect the late processing of the affective value of feedback stimuli [18], [27], [28]. The above-mentioned P2 and LPP findings in ADHD therefore suggest normal early attentional selection of performance feedback, but deviant late processing of negative feedback.

These findings would fit with the above-described models predicting that the detection of external feedback is relatively intact [10], [17]. Moreover, as the children with ADHD in our previous study differed from the TD children with respect to the learning effects on the ERP components, the results can additionally be explained by the model predicting that the consequences of reinforcement are not implemented in the child’s reinforcement history [10]. Whereas the TD children demonstrated a reduction in the feedback P2 amplitude and an increase in self-monitoring at the time of the response (as measured with the response-locked error Positivity), the children with ADHD did not show these learning effects. This was interpreted as a deficit in shifting from feedback (external) monitoring to response (internal) monitoring while learning from external feedback [18]. This learning deficit, however, was found only for a group of children with ADHD that was free from stimulant medication (Mph) during the experiment. Another ADHD group that was kept on medication did not show this deficit, but like their Mph-treated peers showed an overall reduced LPP to negative feedback.

Given the different predictions of the motivational models of ADHD for reward and punishment sensitivity, it is an intriguing question how reward and punishment might influence the monitoring of performance feedback in children with ADHD. Recently, Van Meel and colleagues [28] investigated ERPs reflecting the early detection and late processing of performance feedback coupled with monetary reward and punishment. The early feedback detection was measured with the Feedback Related Negativity (FRN) which has previously been associated with the operation of a monitoring system that calls for additional control whenever an outcome is aversive and/or unexpected [28], [29]. The late processing of the affective value of feedback was measured with the LPP. In that study, a time production task was used, in which positive feedback was given when the participant produced a reaction time of 1 second that fell within a specified time window and negative feedback whenever it fell outside that window. In different conditions, feedback was coupled with monetary gains, losses or no incentives. In TD children, omitted gains as well as omitted losses evoked an FRN, which was absent in children with ADHD. This was regarded as evidence that children with ADHD suffer from a deficient detection of motivationally significant cues. According to the authors, the LPP findings suggested a failure to assign sufficient attention to the emotional impact of negative events such as punishment, but oversensitivity to the loss of desired rewards in children with ADHD.

The finding of absent FRN responses to omitted losses or omitted gains in children with ADHD is in line with previous Evoked Cardiac Response (ECR) studies. At least four studies found that heart rate decelerations of children with ADHD are less responsive to performance feedback stimuli or discriminate to a lesser extent between positive and negative feedback compared to controls [30], [30]–[33]. In a previous ECR study by our group [30] children performed a selective attention task in a condition without performance feedback, a condition with performance feedback coupled with monetary gains, and a condition with monetary losses. In TD children, all conditions elicited a heart rate deceleration on error trials with negative feedback, which was absent in children with ADHD. This suggested that children with ADHD are autonomically less responsive to different types of aversive events, such as error commission, punishment and loss of reward. Interestingly, this held for only the group of children that was free from Mph medication during the experiment. Another group taking Mph demonstrated similar heart rate decelerations as the TD group in the condition without performance feedback and the punishment condition. This suggested that Mph has a stimulating effect on self-monitoring of errors as well as on punishment processing [30].

In the present study we further examined the monitoring of performance feedback coupled with monetary gains and losses in children with ADHD and the modulating effects of Mph. To this end we analysed the feedback-related ERPs that were collected during the ECR study [30]. A group of TD children and two groups of age and intelligence matched children with ADHD, a Mph-free group and an Mph-treated group, performed a selective attention task without performance feedback (*no feedback condition*), with performance feedback coupled to gains (*gain condition*), and with performance feedback coupled to punishment (*loss condition*). Regarding the medication-free children with ADHD we expected that they would show an intact early attentional selection of feedback as reflected by a normal feedback P2, but deviant early feedback detection as reflected by a reduced or absent FRN. Based on the outcomes of the study by Van Meel and colleagues [28] we also expect deviant late processing of the affective value of error feedback, as should be reflected by a reduced LPP to losses in the loss condition in children with ADHD, but an increased LPP to gain omission in the gain condition. Regarding the Mph-treated children with ADHD we expected ‘normalization’ of these deficits, because beneficial effects have been demonstrated on ECR-measures of performance feedback monitoring, especially in the loss condition [30]. Because ADHD is a heterogeneous disorder, with large individual differences in the type, number and severity of the symptoms we also explored correlations between the ERP components and ADHD behaviour questionnaire scales (parent and teacher report). Importantly, previous studies have indicated that internal error monitoring and external feedback monitoring are interdependent; It has been demonstrated that when sufficient information about the performance is present at the time of the response, an Error Related Negativity (ERN) occurs directly after the error response and that the FRN is small or even absent at the time of the feedback [34], [35]. As possibly aberrant error monitoring in ADHD (see for a review [36]) might influence the processing of reward and punishment, we also analysed ERPs time-locked to the response.

## Methods

### Ethics Statement

This study was approved by the Medical Ethical Committee of the University Medical Center Groningen. Written informed consent was obtained from all parents and from 12-year-olds.

### Participants

This study included 47 children belonging to three experimental groups: a control group with TD children (n = 17), an Mph-free ADHD group (n = 15) and an Mph-treated ADHD group (n = 15). In total 50 children had been tested, but three of them had to be removed because of unreliable ERP-data. The data of the total sample of 50 subjects regarding task performance and feedback-related heart rate changes have been published previously [30]. The TD children were recruited from primary schools in the city of Groningen and by advertisement in the newsletter of the University Medical Center in Groningen (UMCG).

The inclusion criteria for all children were: 1) 10 to 12 years of age, 2) a full-scale Intelligence Quotient (IQ) over 80 as measured by the Wechsler Intelligence Scale for Children-III (WISC-III), 3) right handedness (or a tendency to right handedness). Handedness was measured by a self-report list [37]. The TD children were not allowed to have a psychopathological diagnosis or suspicion for ADHD or behavioural problems. The presence of psychopathology was checked by means of the Child Behavioural Checklist which was completed by the parents of all children [38]. None of the TD children scored within the clinical range of the subscales, including the attentional problems subscale which is a screener for ADHD-symptoms, or the total problem scale of the CBCL, with the exception of one TD girl scoring within the clinical range of the internalizing subscale. See Table 1 for an overview of the group characteristics.

**Table 1:** Group characteristics.

	TD (n = 17)	Mph-treated ADHD (n = 15)	Mph-free ADHD (n = 15)	
	Ratio	Ratio	Ratio	p (χ2)
Handedness (ratio: left/ambidexter/right)	0/4/13	0/1/14	0/2/13	ns
Gender (ratio: male/female)	12/5	14/1	13/2	ns
Mph intake in past year (ratio: on/off)	0/17	14/1	11/4	ns
**Measures**	**Mean (SD)**	**Mean (SD)**	**Mean (SD)**	**p (ANOVA)**
Age (years)	11.5 (1.0)	11.4 (0.8)	11.7 (0.8)	ns
Total IQ	103 (9.7)	97 (11.6)	100 (13.5)	ns
Verbal IQ	107 (10.5)	99 (13.0)	101 (9.9)	ns
Performance IQ	97 (13.1)	97 (12.7)	98 (17.4)	ns
DISC Attentional Problems	_	12.3 (5.1)	13.1 (3.5)	ns
DISC Hyperactive Impulsive Behaviour	_	13.0 (2.8)	12.5 (5.1)	ns
CBCL Total Problems	15.0 (11.8)	47.5 (27.2)	62.5 (18.7)	<.001 (TD< Mph-free & Mph-treated ADHD)
CBCL Attentional Problems	2.3 (2.1)	9.6 (3.4)	11.4 (1.7)	<.001 (TD< Mph-free & Mph-treated ADHD)
CBCL Internalizing Problems	4.5 (4.5)	8.5 (8.3)	12.1 (8.3)	<.05 (TD<Mph-free ADHD, Mph-free ADHD = Mph-treated ADHD)
CBCL Externalizing Problems	3.4 (3.6)	13.4 (7.7)	18.5 (6.4)	<.001 (TD< Mph-free & Mph-treated ADHD)
CTRS-R Oppositional	_	59.1 (10.4)	59.9 (13.9)	ns
CTRS-R Inattentive/Cognitive Problems	_	55.2 (8.4)	58.7 (12.8)	ns
CTRS-R Hyperactivity-Impulsivity	_	66.7 (9.6)	65.3 (14.1)	ns
CTRS-R Anxious/Shy	_	61.3 (12.6)	65.1 (11.7)	ns
CTRS-R Perfectionism	_	56 (12.5)	53.1 (9.4)	ns
CTRS-R Social Problems	_	57.5 (8.7)	59.1 (16.0)	ns
CTRS-R ADHD index	_	64.1 (7.9)	64.9 (14.7)	ns
SCQ Total	_	6.8 (4.4)	5.0 (1.7)	ns

Note: TD = Typically Developing, DISC = Diagnostic Interview Schedule for Children, CBCL = Child Behavioural Checklist, CTRS-R = Conners’ Teacher Rating Scale- Revised, SCQ = Social.

The children with ADHD had to meet the criteria of the DSM-IV-TR diagnosis ADHD of the combined type without comorbid internalizing, externalizing and autistic spectrum disorders [1]. All children with ADHD had been diagnosed by independent child psychiatrists of the Department of Child- and Adolescent Psychiatry of the UMCG. This diagnosis was checked by administering the ADHD section of the Diagnostic Interview Schedule for Children to the parents [39], [40] and the Conners’ Teacher Rating Scale-Revised (CTRS-R) to the teachers of the children with ADHD [41], [42]. All children with ADHD scored in the clinical range of the DISC-IV ADHD section or at least in the borderline range of the CTRS-R. As 26 of the 30 children with ADHD were Mph-responders, medication-intake in the period in which the ADHD interview was performed, likely caused underreport of ADHD symptoms. However, the Mph-treated and Mph-free ADHD group did not differ in the number of symptoms as measured by the DISC-IV (see Table 1).

Of the 30 children with ADHD, 26 children were Mph-responders, who had all taken this drug during the main part of the year preceding the experiment (except for one boy who had started the treatment two months before the experiment). The four remaining children with ADHD were freshly diagnosed and not yet referred for pharmacological treatment, and were directly assigned to the Mph-free condition. The Mph-responders were randomly assigned to the Mph-treated (n = 15) or Mph-free condition (n = 15). Those assigned to the Mph-free condition were asked to discontinue Mph-intake for at least 17 hours before they entered the experiment. Of the 30 children with ADHD 13 children scored within the clinical range of the externalizing scale of the CBCL (see Table 1). Even though externalizing disorders were an exclusion criterium for the study, some children with ADHD were reported by their parents to show symptoms of Oppositional Defiant Disorder or Conduct Disorder. The Mph-free and Mph-treated ADHD groups, however, did not differ in the amount of parent reported externalizing problems (see Table 1).

The children in the ADHD groups were screened for autistic spectrum disorder symptoms by their score on the Social Communication Questionnaire reported by the parents [43], which is a screening tool for ASD based on the Autism Diagnostic Interview-Revised [44]. All children with ADHD scored below the cut-off, except for one boy in the Mph-treated group who scored on the cut-off of 15. The two ADHD groups did not differ with respect to autistic type behaviour (see Table 1).

### Task and Procedure

In the selective attention task adopted in this study, the children were asked to sort hierarchical stimuli according to shape and while doing so to earn as much money as possible. The hierarchical stimuli consisted of one large geometric figure (circle, square or triangle), which was built up from smaller geometric figures (circles, squares or triangles). See reference [30] for a more detailed description of the stimulus material. Within one block the stimuli consisted of two possible geometric figures (circles and squares, squares and triangles, or circles and triangles). Each geometric figure was assigned to one of two keys, e.g. the right key should be pressed for a circle and the left for a square. During global blocks, the children were asked to attend only to the large figures and during the local blocks the children were asked to attend only to the small figures. The stimulus sets of the global and local blocks were identical. The hierarchical figures could be congruent (50% of the trials), i.e. the required response is equal for both levels, or incongruent (50% of the trials), i.e. the required response for the attended level is opposite to the one required for the unattended level. Congruent figures for example consisted of a large circle composed of smaller circles, while an incongruent circle for example consisted of a large circle composed of smaller squares. The children performed six global and six local blocks, each consisting of 80 trials and four ‘warming-up’ trials at the start.

Each trial started with a stimulus presentation of 100 ms, followed by a fixation cross with a fixed duration of 1150 ms. The feedback stimulus was time locked to the stimulus and had a duration of 1000 ms. A variable Inter Trial Interval of 500 or 750 ms was adopted. The trial duration ranged from 2.75–3 s. The stimulus presentation in the task was machine-paced. To take individual differences in response speed into account, individual deadline times were calculated for each subject, which was done separately for global congruent and incongruent trials as well as for local congruent and incongruent trials. These individual deadline times (mean reaction time in one condition +10%) were determined in one local and one global deadline determination block preceding the experimental blocks. The response window ran from stimulus onset until the end of the individual deadline time (which could differ between the stimulus types), but the time between stimulus onset and feedback onset endured (100+1150 = ) 1250 ms for each individual. In the experiment, all children were encouraged to earn as much money as possible, but were at the same time forced to react quickly as late reactions resulted in a penalty of 0.02 €.

The 12 blocks were divided into three feedback conditions: no feedback, gain and loss. This resulted in four blocks per feedback condition (320 trials), with each feedback condition containing two global and two local blocks (160 trials each). In the no feedback condition the children received no information about their performance; each response was followed by a question mark. After finishing a no feedback block the children received 0.70 € independent of their performance. In the reward condition the children started with 0.00 € and only correct responses resulted in a gain of 0.01 €. Gain and no gain were indicated by ‘+1 c’ (in green) and ‘+0 c’ (in red) respectively. In the punishment condition the children started with 0.80 € and only incorrect responses resulted in a loss of 0.01 €. Loss and no loss were indicated by ‘−1 c’ (in red) and ‘−0 c’ (in green) respectively. Trials with late responses were indicated by ‘too late’ (in black) and resulted in a loss of 0.02 €. After every block the children received the money they had earned from the experimenter in the form of coins. See Table 2 for an overview of the feedback conditions.

**Table 2:** Description of the feedback conditions.

	No feedback	Gain	Loss
Start amount	70 c	0 c	80 c
Maximum amount	70 c	80 c	80 c
Correct trial	?	+1 c	−0 c
Incorrect trial	?	+0 c	−1 c

Note: c = cents.

The children were seated on a comfortable chair in front of a computer screen in a room that was separated from a control room by a one-way screen. After a standardised instruction the children performed four short practice blocks consisting of 20 trials each, first a global and local block with unlimited stimulus duration and second a global and local block with short (100 ms) stimulus presentation (∼10 min). This was followed by two deadline blocks consisting of 80 trials each (∼10 min), in which the individual deadline for each stimulus type was calculated. After application of the electrodes the children performed the twelve experimental blocks (each lasting ∼5 minutes), with a total task duration of ∼60 minutes. Between each block a break of a few minutes was taken, in which the child received payment. After six experimental blocks there was a break of ∼20 minutes.

### EEG Event Related Potentials

The EEG was recorded using a lycra stretch cap (Electro-Cap Center BV) with 21 electrodes, placed according to the 10–20 system (O1, Oz, O2, P3, P5, P7, Pz, P4, P6, P8, C3, Cz, C4, F3, Fz, F4, F7, F8, FP1, FPz en FP2). Vertical and horizontal eye movements were recorded with electrodes respectively above and next to the left eye. For all channels Ag-AgCl electrodes were used and impedances were kept below 10 kΩ. Using the REFA-40 system (TMS International B.V.) all channels were amplified with filters set at a time constant of 1 second and a cut-off frequency of 130 Hz (low pass). The data from all channels were recorded with a sampling rate of 500 Hz using Portilab (version 1.10, TMS International B.V.). Using BrainVision 2 (Brain Products), the signals were off-line filtered with a 0.10 Hz high pass and 35 Hz low pass filter, and referenced to the left ear electrode.

To investigate the FRN, P2 and LPP, EEG segments were computed from 200 ms before to 1000 ms after feedback onset, with the first 200 ms serving as a baseline. Separate segments were computed for the correctness of feedback, i.e. correct and incorrect feedback. Trials with late responses were excluded. For the ERN and Pe, segments were computed around the responses ranging from 500 ms before to 800 ms after response onset, with the first 200 ms serving as a baseline. This was done for both response types, i.e. correct and incorrect responses.

Segments containing artefacts were excluded from further analysis, i.e. segments exceeding a voltage difference of 200 µV on central and posterior electrodes and 300 µV on frontal electrodes, segments with low activity and segments with spikes. Segments with eye movements and blinks were kept and corrected, adopting the standard Gratton & Coles ocular correction procedure [45]. Beforehand EOG segments containing artefacts exceeding 500 µV/100 ms or low activity were removed. Children showing more than 40% of data loss in any condition were manually checked for artefacts with somewhat wider criteria for the EEG and/or EOG (50–100 µV higher for the maximum allowed voltage difference in the segments). Thirty-four children showed eye blink artifacts exceeding 300 µV in the frontal EEG and 6 children showed slow wave activity exceeding 200 µV at central or posterior EEG. Feedback-locked segments including eye blinks or movements preceding 200 ms post feedback were removed. From the original sample, 2 boys with ADHD (one on and one off Mph) had to be removed from analysis, because their EEG data contained excessive artefacts. One girl from the TD group had to be removed from analysis, because too few error trials could be included in the average ERP.

For every feedback condition, the response locked ERPs contained on average 219 (SD 51) trials for correct responses and 49 (SD 25) for incorrect responses. This was similar in the feedback locked ERPs, with 208 (SD 47) trials for correct and 46 (SD 25) for incorrect trials. Across feedback conditions, the ADHD groups did not differ significantly from the TD group in the number of trials for ERPs of correct or error responses (one-way ANOVA’s respectively: *F*(2,46) = 1.1, *p*>.05; *F*(2,46) = 1.6, *p*>.05), or ERPs of positive or negative feedback (respectively: *F*(2,46) = 1.9, *p*>.05; *F*(2,46) = 0.8, *p*>.05).

Individual averages were calculated for the 21 electrode positions and three feedback conditions and two response types (correct and incorrect). The averages were collapsed for the global and local blocks, because the separate averages for these conditions contained too few error trials for reliable averages.

### Data Analyses

#### Performance measures

The percentage of correct responses and correct RTs were analysed by means of a 3*2 mixed ANOVA design (SPSS version 16.0) with the within subject variables ‘feedback’ (no feedback, gain and loss) and ‘level’ (global, local) (accession number for the publicly available database will be provided during review). Simple contrasts were computed for the factor feedback. Trials with late responses were excluded from the analysis.

#### ERP measures

A frontocentral feedback P2 peaked around 200 ms after feedback onset (see for topographical maps Figure S1), which is consistent with previous studies [18], [21]. This peak was quantified as the averaged amplitude of a 50 ms interval ranging from 170–220 ms on Fz. After the P2, a negativity was observed over frontocentral regions (see for topographical maps Figure S2). In the conventional time interval of 200–400 ms of the FRN [35], [46] after feedback onset difference waves of correct minus incorrect trials indicated only small FRN amplitudes in some conditions (see for topographical maps Figure S3). The largest differences were present in the interval of 260–360 ms, which is in agreement with previous FRN latency findings in children [28]. The FRN was quantified as the averaged amplitude of a 100 ms interval ranging from 260–360 ms on Fz. From 450 ms onwards a feedback LPP developed with a widespread centroparietal topography (see for topographical maps Figure S4), which is consistent with previous studies [18], [28]. The LPP was quantified as the mean averaged amplitude of a 350 ms interval ranging from 450–800 ms on Pz.

To check for group differences in response monitoring, the response-locked ERN and Pe amplitude were analysed. Consistent with previous studies in children [47], [48], the ERN peaked early around response onset at frontocentral electrodes. This peak was quantified as the averaged amplitude of a 200 ms interval ranging from −100–100 ms on Fz. The Pe emerged from 100 ms after response onset over centroparietal electrode positions. This peak was quantified as the averaged amplitude of a 300 ms interval ranging from 100–400 ms on Pz.

The ERP measures were analysed by means of a 3*2*3 repeated measures ANOVA design with the within subject variables ‘feedback condition’ (no feedback, gain and loss) and ‘response type’ (correct vs. incorrect) and ‘group’ as the between subjects variable (TD, Mph-free ADHD, Mph-treated ADHD) (accession numbers for the publicly available databases will be provided during review). Note that positive and negative feedback are referred to as respectively ‘gain’ and ‘no gain’ in the gain condition, and ‘no loss’ and ‘loss’ in the loss condition. In all analyses, main effects of group and interactions with group were specified for significant effects (p<.05). Group differences were analysed by means of three post hoc pairwise group comparisons: TD vs. Mph-treated ADHD, TD vs. Mph-free ADHD, Mph-free vs. Mph-treated ADHD. Greenhouse-Geisser adjusted p-values and the epsilon correction factor are reported for within subject factors with more than two levels, with the unadjusted degrees of freedom and F-values. Partial eta squared effect sizes (η^2^) are reported. In order to check whether internalizing and externalizing problems and gender confounded the group effects, all analyses were repeated with the CBCL internalizing and externalizing subscales and gender as covariates.

#### Correlational analyses

For the exploration of associations between ADHD-symptoms and the feedback monitoring ERP components, Pearson correlations were computed between the feedback-related ERP-amplitudes and scale scores of ADHD questionnaires. Three teacher-reported CTRS-R scales were included: ‘Inattentive/Cognitive problems’, ‘Hyperactive-Impulsivity’ and ‘ADHD index’ and two parent-reported scales were included: the DISC-IV scales ‘Attentional problems’ and ‘Hyperactive Impulsive Behaviour’. Correlations were computed with the mean amplitudes of the feedback P2 and LPP for gain, no gain, loss and no loss trials, and the FRN difference amplitudes of correct minus incorrect trials for the gain and loss condition (accession number for the publicly available database will be provided during review). Significant correlations (p<.05) were checked for outliers and significant correlations were only reported when outliers could not explain the correlation. In order to check whether internalizing and externalizing problems and gender influenced the correlations, partial correlations were computed with the CBCL internalizing and externalizing subscales and gender as control variables.

## Results

### Performance Measures

#### Deadlines and late reactions

The groups did not differ significantly in duration of the mean individual deadline, but for all groups the mean individual deadline was shorter in the global than in the local condition (Mean (SD): global = 734 (144) ms; local = 772 (145) ms), which is reflected by a main effect of level (F(1,44) = 10.0, p<.01, η^2^ = .19).

The groups differed significantly in their mean percentage of late responses (*F*(2,44) = 3.5, *p*<.05, *η^2^* = .14). Post hoc group comparisons revealed that the TD group showed less late reactions than the ADHD groups (Means (*SD*): TD = 8% (2,6); ADHD Mph = 10% (4,1) ); ADHD: 11% (4,0)). For all groups the mean percentage of late responses was larger in the global condition than in the local, which is reflected by a main effect of level (*F*(1,44) = 10.8, *p*<.01, *η^2^* = .20) and absence of an interaction with group (p>.05). Moreover, all groups had a higher percentage of late reactions in the no feedback (11%) condition than in the gain and loss conditions (9%), which is expressed by a main effect of feedback (*F*(2,88) = 9.4, *p*<.01, *η^2^* = .18, *ε* = .81) and absence of an interaction with group (*p*>.05).

#### Accuracy

All children were capable of performing well above chance level. The TD group tended to be more accurate on the task than the two ADHD groups (84% vs. 78% respectively). The main effect of group did not reach significance but showed medium effect size (*F*(2,44) = 2.4, *p* = .11, *η^2^* = .10), and group comparisons of the ADHD groups with the TD group revealed trends to significance (TD vs. ADHD Mph: *p* = .07; TD vs. ADHD: *p* = .06). Using the CBCL internalizing subscale score as a covariate strengthened these trends, but the CBCL externalizing subscale score and gender slightly reduced these trends.

**Figure 1:**
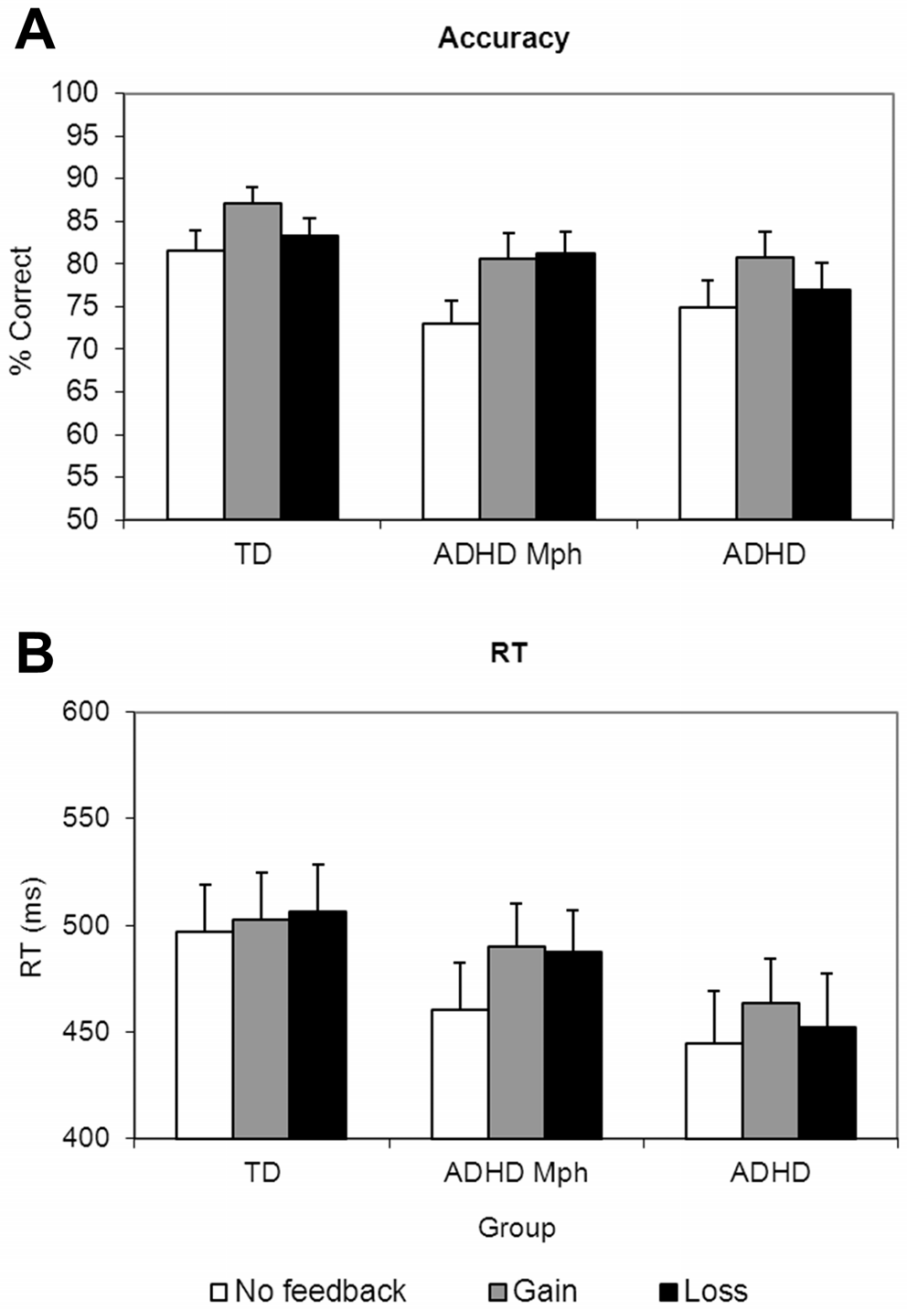
Performance measures. Mean accuracy (A) and reaction time (RT) (B) in the no feedback, gain and loss condition, separated for the three groups. Error bars indicate standard errors.

Regarding the feedback conditions, all groups performed at a lower accuracy level in the no feedback condition compared to the conditions with feedback (see Figure 1). This is reflected by a main effect of feedback (*F*(2,88) = 14.8, *p*<.001, *η^2^* = .25, *ε* = .70), absence of an interaction with group (*p*>.05) and significant contrasts for the factor feedback showing that the no feedback condition differed significantly from the other feedback conditions (no feedback vs. gain: *p*<.001; no feedback vs. loss: *p*<.01). Additional contrasts indicated that the gain condition was superior to the loss condition (gain vs. loss: *p*<.01). Only for this contrast an interaction with group was present (gain vs. loss: feedback*group: *p*<.05). Post hoc pairwise group comparisons indicated that only for the Mph-treated ADHD group the gain condition was not superior to the loss condition. The reported feedback effects did not differ between the global and local condition. Testing for the covariates CBCL internalizing and externalizing subscale scores and gender, revealed that all covariates slightly reduced this group effect.

#### RT

The groups did not differ in mean RT (Mean (SD): 479 (89) ms) and mean RT did not differ for the global and local blocks. Nor did the groups differ in their effect of level. This is reflected by the absence of a main effect of group (*p*>.05), level (*p*>.05) and an interaction of these variables (*p*>.05). As can be seen in Figure 1, all groups responded faster in the no feedback condition than in the conditions with feedback. This is reflected by a main effect of feedback (*F*(2,88) = 5.8, *p*<.05, *η^2^* = .12, *ε* = .69), significant feedback effects for only the contrasts of no feedback vs. gain (*p*<.05) and no feedback vs. loss (*p*<.05) and the absence of any interaction between feedback and group. None of the reported feedback effects interacted with the factor level. Using the CBCL internalizing and externalizing subscale scores and gender as a covariate did not change the group effects.

### Feedback-locked ERPs

#### Feedback P2

The Feedback P2 on Fz in the interval of 170–220 ms differed significantly between feedback conditions, which was reflected by a significant main effect of feedback condition (*F*(2,88) = 24.4, *p*<.001, *η^2^* = .36, *ε* = .99) and interaction of feedback condition*response type (*F*(2,88) = 8.7, *p*<.001, *η^2^* = .17, *ε* = .96). Contrasts indicated that the Feedback P2 in the no feedback condition was decreased compared to the gain and loss condition (no feedback vs. gain; *p*<.001; no feedback vs. loss: *p*<.001; gain vs. loss: *p*>.05). The *no feedback* condition neither contained a significant effect of response type, nor an effect of or interaction with group.

**Figure 2:**
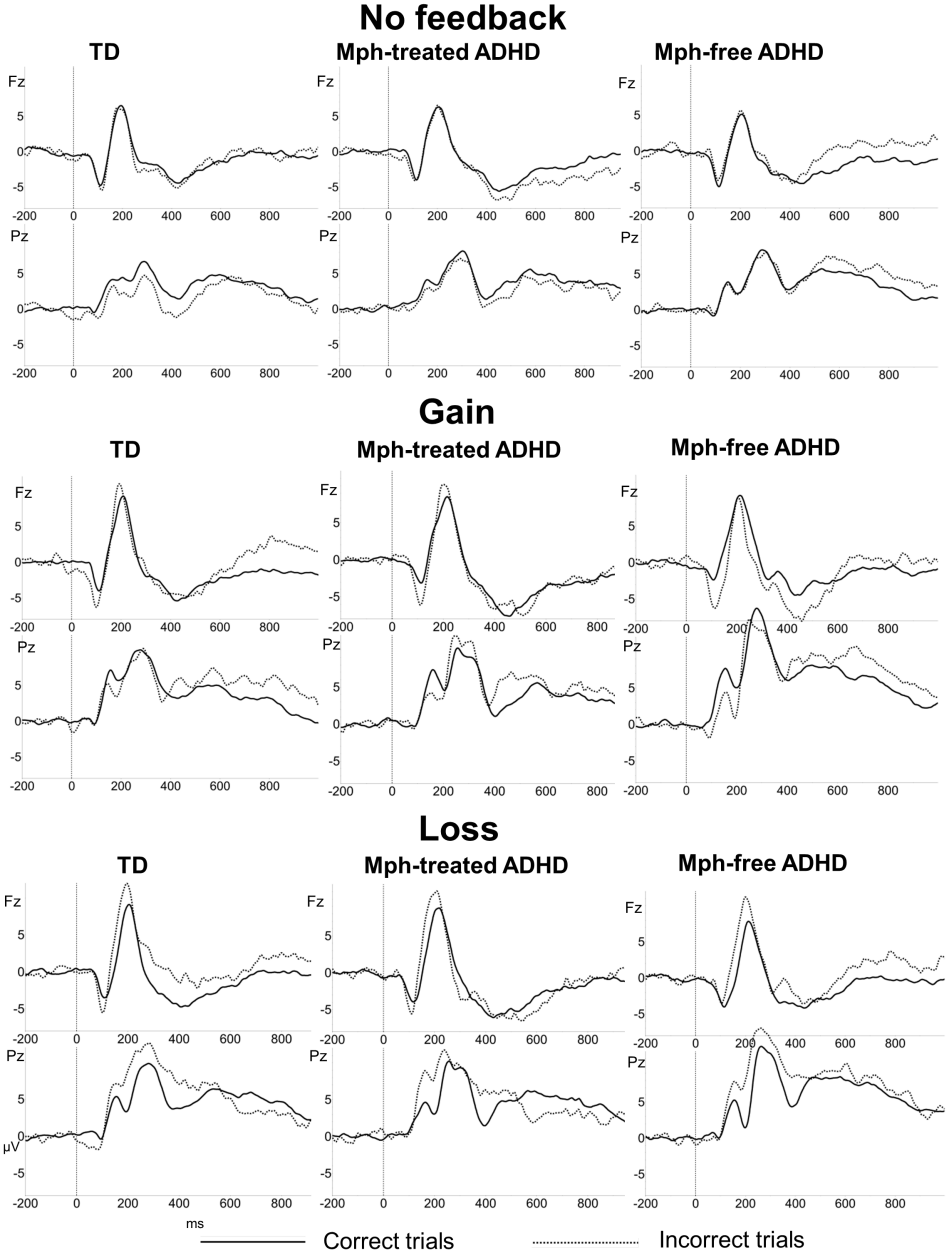
Feedback-locked ERPs. Grand average feedback-locked ERPs of the typically developing (TD), Mph-treated ADHD and Mph-free ADHD group, elicited by feedback stimuli on correct trials (solid lines) and incorrect trials (dashed lines) separated for the three feedback conditions (no feedback, gain and loss).

**Figure 3:**
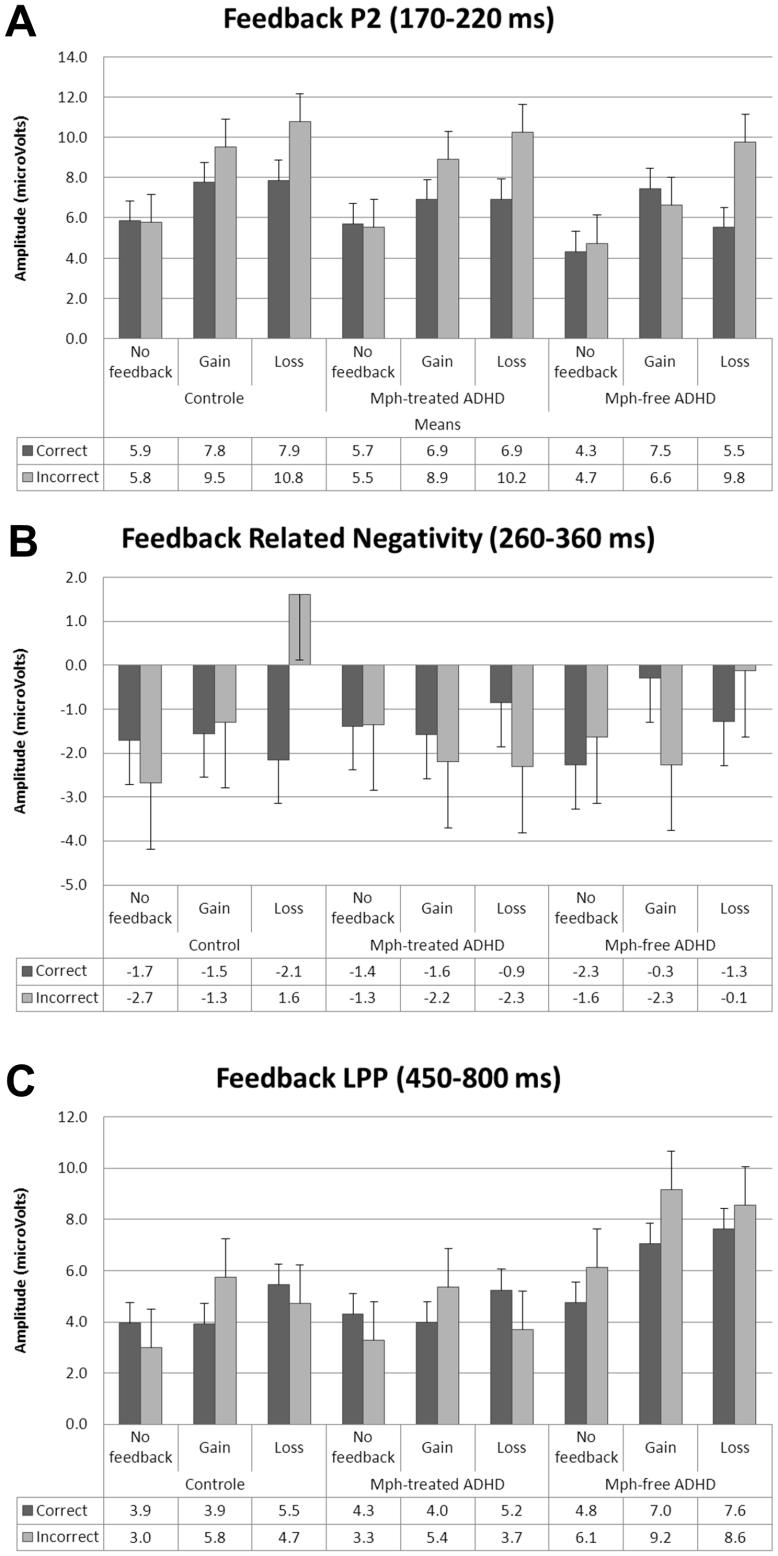
Bar graphs of the feedback-locked ERP amplitudes. Mean amplitudes of the Feedback P2 (A), Feedback Related Negativity (FRN) (B), and feedback Late Positive Potential (C) of the typically developing (TD), Mph-treated ADHD, and Mph-free ADHD groups for correct and incorrect trials in the three feedback conditions (no feedback, gain and loss). Error bars indicate standard errors.

Further analyses were conducted with the factor feedback (gain/loss) excluding the no feedback condition. The gain and loss condition differed with respect to the response type effect, as reflected by an interaction of feedback (gain/loss)*response type (*F*(1,44) = 7.2, *p*<.05, *η^2^* = .14). Only in the *loss condition* a significant effect of response type was present (*F*(1,44) = 25.6, *p*<.001, *η^2^* = .37), indicating that the Feedback P2 was increased in amplitude for loss trials (i.e. –1 cent) compared to no loss trials (i.e. –0 cent), see Figure 2 and 3. These effects did not differ between groups (*p*>.05). Adding the CBCL internalizing and externalizing subscale scores and gender as covariates did not alter the ns group effects.

#### FRN

As can be seen in Figure 2 and Figure S3 of the Appendix, no typical FRN peak was elicited on incorrect trials on frontocentral electrode positions in the expected latency range of 200–400 ms. Analyses in the interval of 260–360 ms on Fz revealed no significant effects of response type, feedback or an interaction or response type*feedback (*p*>.05), suggesting that across groups the FRN was not sensitive to the type of feedback. However, a feedback*response type*group effect was present (*F*(4,88) = 2.7, *p*<.05, *η^2^* = .11, *ε* = .82), which was only significant for the contrast of no feedback vs. loss (*F*(2,44) = 4.8, *p*<.05, *η^2^* = .18) and not for the other contrasts (no feedback vs. gain, *p*>.05; gain vs. loss, *p*>.05). As can be seen in Figures 2 and 3, the TD group showed a positive ERP amplitude to losses in the FRN interval. Post hoc group comparisons revealed that only the Mph-free ADHD group differed significantly from the TD group for this contrast (*F*(1,30) = 4.8, *p*<.05, *η^2^* = .14), with TD children showing a more positive potential for losses than the Mph-free ADHD group. Adding the CBCL internalizing and externalizing subscale scores as covariates reduced these group differences to trends. The covariate gender did not alter the group effects.

#### Feedback LPP

The LPP amplitude over Pz was increased in the gain and loss condition compared to the no feedback condition, see Figures 2 and 3. This was reflected by a main effect of feedback (*F*(2,88) = 6.0, *p*<.01, *η^2^* = .12, *ε* = 1.0) and significant contrasts, indicating that the no feedback condition differed from the gain and loss condition (no feedback vs. gain; *p*<.01; no feedback vs. loss: *p*<.01; gain vs. loss: ns). Further analyses were again conducted with the factor feedback (gain/loss) excluding the no feedback condition. Independent of group, the LPP amplitude was enhanced for no gain trials in the gain condition only. This was reflected by an interaction of feedback (gain/loss)*correctness (*F*(1,44) = 4.6, *p*<.05, *η^2^* = .10), a main effect of correctness in the gain condition (*F*(1,44) = 5.5, *p*<.05, *η^2^* = .11) but not in the loss condition (*p*>.05), and absence of an interaction with group.

Independent of feedback condition (gain/loss), the Mph-free ADHD group compared to the TD and Mph-treated group, showed enhanced LPP amplitudes to the feedback stimuli. This was reflected by a main effect of group (*F*(2,44) = 4.5, *p*<.05, *η^2^* = .17) and significant post hoc group comparisons (TD vs. Mph-free ADHD: *F*(1,30) = 5.6, *p*<.05, *η^2^* = .16; Mph-free vs. Mph-treated ADHD: (*F*(1,28) = 10.3, *p*<.01, *η^2^* = .27). In the no feedback condition no group effects/interactions were present. Adding the CBCL externalizing subscale score as a covariate reduced this group difference to a trend with medium effect size (*F*(2,43) = 2.9, *p* = .065, *η^2^* = .12). The covariates CBCL internalizing subscale score and gender did not alter the group effects.

### Response-locked ERPs

#### ERN

**Figure 4:**
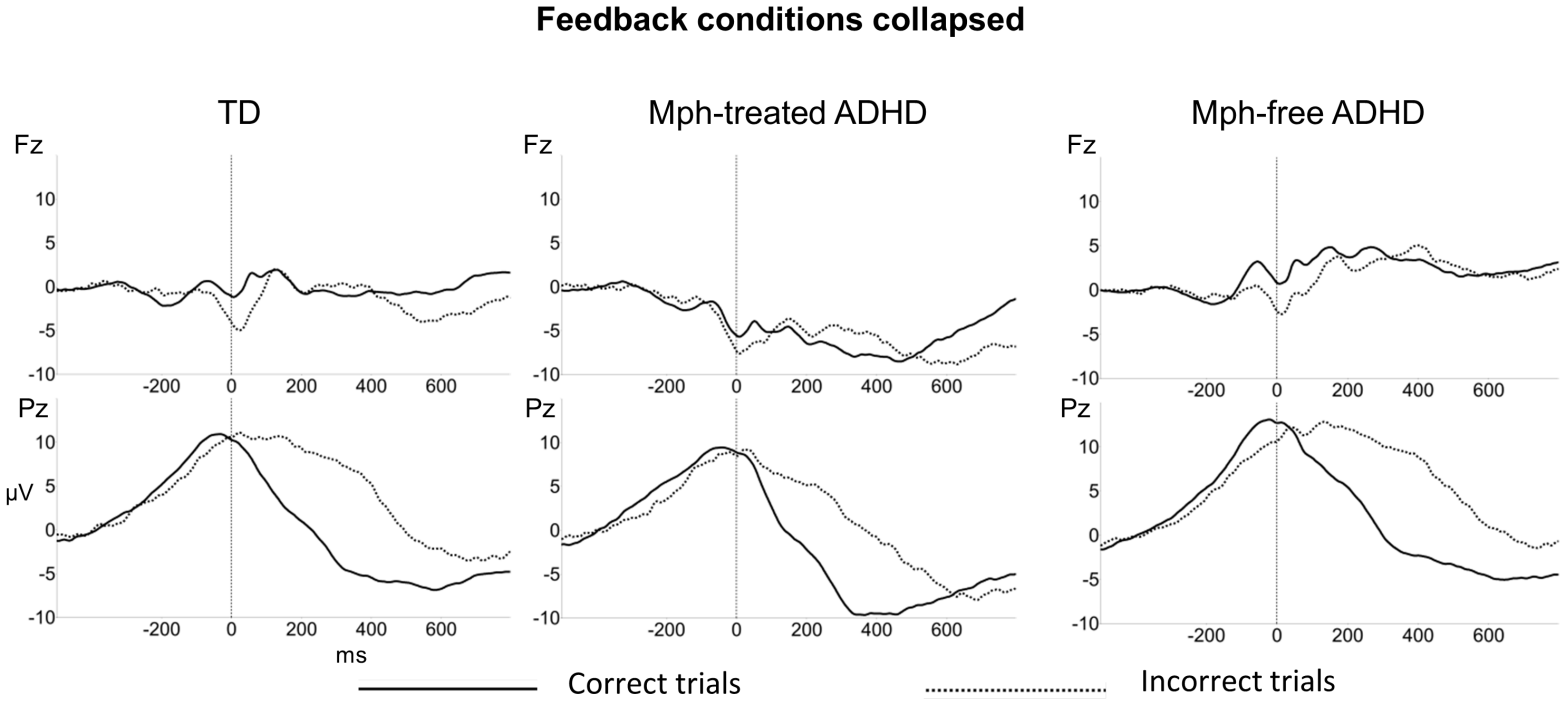
Response-locked ERPs. Grand average response-locked ERPs of the typically developing (TD), Mph-treated ADHD and Mph-free ADHD group, elicited by correct responses (solid lines) and incorrect responses (dashed lines) collapsed across the three feedback conditions. The feedback conditions were collapsed because no interactions of feedback type with response type were present.

The ERN on Fz in the interval of −100 to 100 ms revealed a significant effect of response type (*F*(1,44) = 22.6, *p*<.001, *η^2^* = .34), indicating that the ERN amplitude is more negative for incorrect than correct trials, see Figure 4. There were no significant effects of feedback, interactions of response type with feedback condition or group and neither was there a main effect of group (all *p*>.05). Adding the CBCL internalizing and externalizing subscale scores and gender as covariates did not alter the ns group effects.

#### Pe

The Pe on Pz in the interval of 100 to 400 ms revealed a significant effect of response type (*F*(1,44) = 222.0, *p*<.001, *η^2^* = .84), indicating that this positivity is larger for error trials than correct trials. No significant interactions with group or feedback condition were present. A significant overall group effect was present (*F*(2,44) = 4.8, *p*<.05, *η^2^* = .18). Post hoc group comparisons indicated that the Mph-treated ADHD group showed a less positive potential across correct and incorrect trials than both the Mph-free ADHD group and the TD group (ADHD Mph vs. ADHD: *F*(1,28) = 8.5, *p*<.01, *η^2^* = .23; ADHD Mph vs. TD: *F*(1,30) = 3.0, *p* = .09, *η^2^* = .09). Adding the CBCL internalizing and externalizing subscale scores and gender as covariates did not alter the ns group effects.

#### Correlations of ERP components with behaviour scales

**Figure 5:**
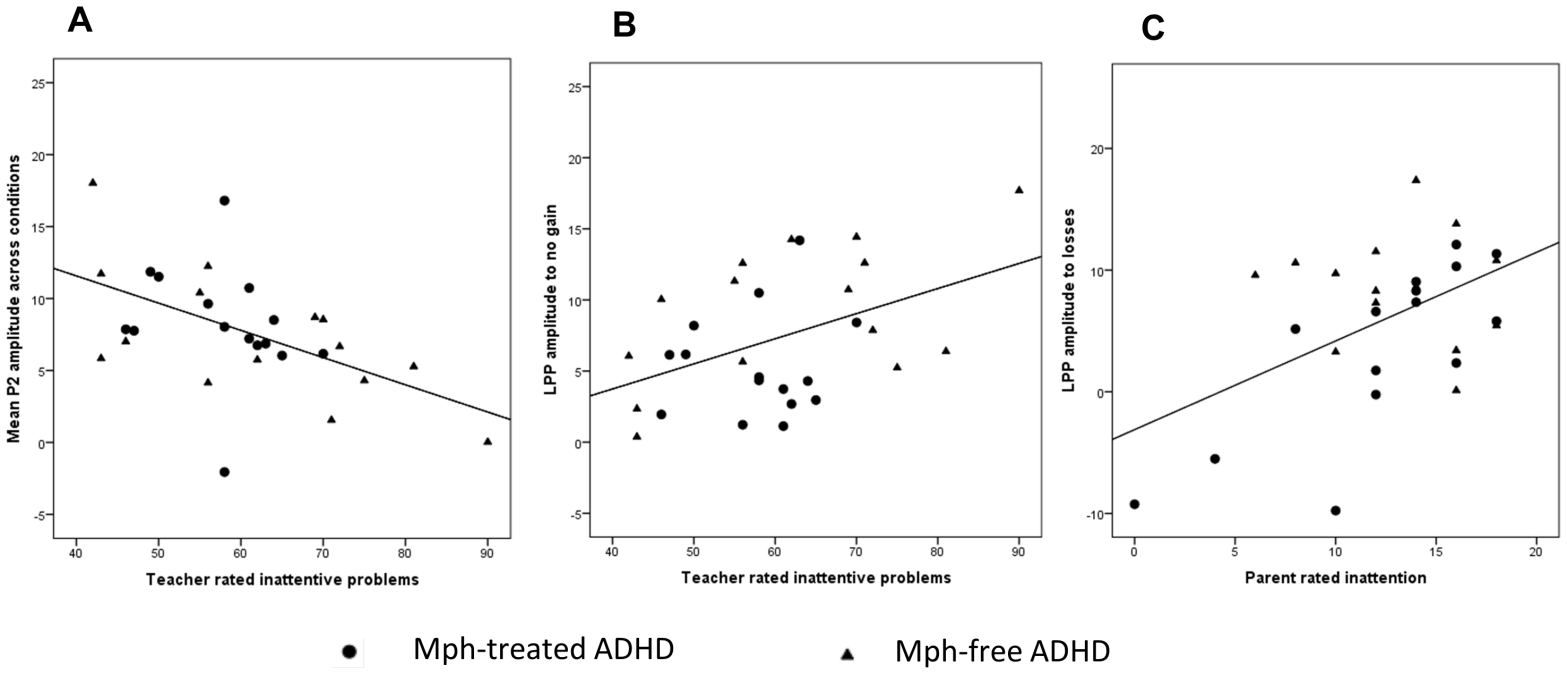
Scatterplots of associations between feedback-locked ERPs and severity of inattention problems. Scatterplots of associations between feedback-locked ERP amplitudes in microvolts and the ADHD inattention scales; A) negative association of teacher rated inattentive problems (CTRS-R) and feedback P2 in gain and loss condition (*r* = –.38 to –.56), B) positive association of teacher rated inattention (CTRS-R) and feedback LPP to no gain (*r* = .44), C) Positive association of parent rated inattention (DISC-IV) and feedback LPP to losses (*r* = .50). The correlations were computed across Mph-treated (circles) and Mph-free (triangles) children with ADHD.

The feedback P2 amplitude to all feedback stimuli correlated *negatively* with teacher rated inattention problems on the CTRS (gain: *r*(30) = –.56, *p*<.01; no gain: *r*(30) = –.44, *p*<.05; no loss: *r*(30) = –.38, *p*<.01; loss: *r*(30) = –.42, *p*<.05), see Figure 5. Deselecting 3 outliers strengthened these correlations. In contrast, the feedback LPP amplitude to no gains in the gain condition was correlated *positively* with teacher rated inattention problems (no gain: *r*(30) = .44, *p*<.05) and the feedback LPP amplitude to losses in the loss condition was correlated *positively* with parent reported inattention problems on the DISC-IV (loss: *r*(30) = .50, *p*<.01). Deselecting 2 outliers did not change these correlations. No other significant correlations, which were not caused by outliers in ERP amplitudes, were found with the ADHD behaviour scales. Computing partial correlations controlling for the CBCL internalizing and externalizing subscale scores and gender did not change this pattern of correlations. In general, the correlations were strongest in the Mph-free group because of greater variation in the questionnaire scores compared to the Mph-treated group.

## Discussion

The main aim of this study was to gain insight into the processing of continuous reward and punishment in children with ADHD and the modulating effects of Mph. The performance data indicated that Mph-treated and Mph-free children with ADHD and children without ADHD benefitted equally well from the provision of performance feedback during the task, compared to a condition without feedback. All groups increased in accuracy and slowed down reaction times when performance feedback was provided, suggesting that accurate responding was more important than speed in the gain and loss condition compared to the no feedback condition. The accuracy level in the gain condition was superior to the loss condition in the Mph-free children with ADHD and TD children. Mph-treated children with ADHD however improved their accuracy equally well in the gain and loss condition, but this group effect weakened when controlling for internalizing and externalizing problems and gender. Overall, the performance findings demonstrate increased motivation to perform accurately on the selective attention task in TD children, Mph-free and Mph-treated children with ADHD when they are provided with continuous reward and punishment, and in general continuous reward was superior to punishment.

In line with our previous study [18], the ERPs demonstrated that continuous reward and punishment elicit similar frontocentral P2 amplitudes in the TD group and the Mph-treated and Mph-free ADHD group, suggesting that on a group level early attentional selection of feedback stimuli is intact in medicated and unmedicated children with ADHD. However, the correlational analyses with the ADHD behaviour scales revealed that those children with the ADHD-combined type with higher levels of teacher-reported inattention problems have lower feedback P2 amplitudes to feedback stimuli in both the gain and loss condition. These children had been rated by their teachers higher on items like ‘forgets things’, ‘avoids mental effort’, ‘lacks interest’, ‘fails to finish’, ‘loses things’, but also ‘poor spelling/reading/arithmetic’. These findings suggest that the more severe these inattention problems are in children with combined type ADHD, the weaker the attentional selection of performance feedback is for these children. Thus, although overall group differences in the feedback P2 appeared to be absent, it cannot be concluded that all children with combined type ADHD have an intact early attentional selection of rewarding and punishing feedback, because this appeared to be related to the severity of inattention problems.

No typical FRN was elicited by error trials in the expected latency range of 200–400 ms. This might be explained by the nature of the feedback. The FRN amplitude reflects the extent to which subjective predictions of gains or losses are violated, with larger FRNs accompanying larger violations of subjective expectancy [49], [50]. The FRN has been typically studied in task paradigms with a gambling component [51], with time estimation [52] or probabilistic feedback [34], in which the stimulus-response sets are uncertain and/or the feedback is random. In a task with clearly defined stimulus-response sets and true performance feedback like in the present study, violations of predictions at the time of feedback presentation are small because errors are already processed at the time of the response. This was demonstrated in the present study by the presence of a response-locked ERN and Pe in all groups and in all conditions with no group differences in these components. As mentioned in the introduction, the FRN might be small or even absent at the time of the feedback when an ERN occurs at the time of an error response [34], [35]. Interestingly, we neither found a typical FRN in our previous study with children while using a feedback-based learning task in which the stimulus-response sets were uncertain at the beginning of the task and in which the ERN and Pe were only small in the beginning of the task [18]. Therefore other factors might also contribute to the occurrence of the FRN such as the saliency or magnitude of the reward and punishment.

Yet, the analyses in the FRN interval did reveal that the TD children elicited a more negative FRN potential to no losses than losses, which is in line with previous studies showing larger FRN amplitudes to omitted losses (which is a positive feedback outcome) than losses (which is a negative feedback outcome) [28], [53]. The TD children might have expected more losses than they actually received. Only the Mph-free children with ADHD did not show this effect in the loss condition, which may be indicative of either difficulties in computing reward prediction errors or more positive expectancies of their outcomes in the face of losses. It must be noted that this effect was slightly reduced when controlled for parent-reported internalizing and externalizing problems, suggesting that comorbid problems partly explain this effect. Nevertheless, the finding is in line with the study by Van Meel and colleagues [28] that demonstrated absence of modulation of the FRN amplitude by motivational conditions in medication-free children with ADHD. Based on our results we can however only speculate about expectancies, because the expectancy of reward or punishment was not manipulated in the present task (as evident by the absence of a typical FRN). Previous studies making use of random utilitarian, and therefore less predictable, feedback demonstrated exaggerated FRN amplitudes following monetary losses in children with ADHD [54], at least when the money was made tangible for these children [55].

In contrast to our expectations that were based on previous studies making use of performance feedback [18], [28], the Mph-free children with ADHD did not show a reduced LPP to feedback indicating losses. Instead the Mph-free ADHD group showed an *enhanced* LPP amplitude to all feedback stimuli with large effect size. This effect was reduced to a trend to significance (p = .065) with medium effect size when we statistically controlled for parent-reported externalizing problems, suggesting that part of this effect can be explained by externalizing problems in the children with ADHD. As previous studies have demonstrated that the LPP is enhanced during affective picture processing, especially when attention is explicitly directed to the emotional content of the picture [26], the enhanced feedback LPP in Mph-free children with ADHD might reflect increased attention to the affective value of the feedback stimuli. We speculate that the children with ADHD attached more value to the feedback stimuli than the TD children, because they are more dependent on external motivators to keep up their performance. This might explain why the provision of appropriate reinforcement in children with ADHD in some studies has a more positive effect than in control children [8]. We moreover speculate that in the children with more severe inattention problems, the enhanced feedback LPP might reflect compensatory late feedback processing for the reduced early attentional selection, because inattention problems correlated negatively with the feedback P2 but positively with the LPP to negative feedback. To our best knowledge, this is the first time that an *enhanced* LPP to feedback stimuli has been reported in children with ADHD. This deviates from our previous study [18] on feedback-based learning in a largely overlapping sample that provided some evidence for a reduced LPP amplitude to negative feedback in medication-free children with ADHD. This deviation is likely due to differences in the task paradigms used and the role of feedback in these paradigms. In the present study, with clearly defined stimulus-response couplings that were well-trained in the children, feedback might be more of a confirmation of the performance or a motivator for maintaining optimal performance. In the previous studies [18], [28], with uncertain stimulus-response couplings, the feedback has a more teaching role. These different functions of feedback in different task paradigms might explain the inconsistent findings. The Mph-treated children with ADHD did not differ in their feedback LPP amplitudes from TD children, suggesting that Mph normalizes the deviant late processing of affective feedback stimuli in children with ADHD.

In literature, negative affective information has been shown to elicit larger LPP responses than positive affective information suggesting a ‘negativity bias’ in processing information, e.g. [56]. In the present study omitted gains elicited a larger LPP amplitude than gains in the gain condition, whereas the LPP had similar amplitudes for losses and omitted losses in the loss condition. This suggests that the children in this study had a ‘larger negativity’ bias for negative feedback outcome in the form of reward omission (compared to reward gain) than in the form of punishment (compared to punishment omission). Overall this pattern did not differ between groups like in the study by Van Meel and colleagues [28] who found evidence for a larger LPP ‘negativity bias’ for omitted rewards in the reward condition in TD children, but for losses in the punishment condition in children with ADHD. Yet, our correlational findings provide some evidence that children with ADHD with more severe inattention problems at home or at school have a larger LPP ‘negativity bias’ for omitted gains (compared to gains) as well as losses (compared to omitted losses). To summarize, the findings on the group level imply that the children in this study had a larger ‘negativity bias’ for processing negative feedback in the form of reward omission than of punishment, but the correlational findings additionally imply that children with the combined type ADHD showing more severe attentional problems have a ‘negativity bias’ for both forms of negative feedback.

For a good understanding of the present findings, they must be placed into a larger theoretical perspective. First, we only addressed performance feedback that was contingent to the response and that was provided continuously. As predicted by some ADHD models [5], [7], [10], only delayed performance feedback may be processed differently in children with ADHD. Moreover, the feedback in the present study was highly predictable. Other studies making use of unexpected feedback reported exaggerated FRNs to monetary losses [54], [55], which is in line with findings of increased orbitofrontal activation following reward delivery in adults with ADHD [57]. Although monitoring of immediate and highly predictable performance feedback might be relatively intact, children with ADHD might insufficiently update their reinforcement history after delayed or unexpected feedback, leaving them unprepared for unexpected outcomes [10], [28], [54]. Indeed, neuroimaging studies in adolescents and adults with ADHD have demonstrated reduced ventral striatal activity during the anticipation of reward in gambling tasks [57]–[59], suggesting a reduced preparatory state for upcoming reward. Neurocomputational models predict that this hypofunctional fronto-striatal dopamine system impairs reinforcement learning (Go learning) [11], [16]. This might however become particularly apparent in learning situations with delayed feedback and/or feedback that is difficult to predict. Future ERP studies could shed more light on the neurobiological sensitivity to feedback of children with ADHD, by making a direct comparison of immediate versus delayed feedback and predicted versus unpredicted feedback in children with and without ADHD.

### Study Limitations

There are some limitations to the interpretation of the findings of this study. First, the characteristics of the sample might have influenced the outcomes. The sample size was small (n = 15 in each ADHD group) which increases the chance for coincidental factors influencing the outcomes, such as outliers. We checked whether the LPP effect could be explained by outliers, which was not the case. Nevertheless it is recommended for future studies to use larger samples. Although an exclusion criterium for the ADHD groups was the presence of a clinically assessed comorbid disorder, this was not checked with structured interviews. Parent ratings indicated the presence of externalizing problems in one third of the ADHD sample. The enhanced LPP effect could partly, however not completely, be explained by externalizing problems. Internalizing problems and gender of the subjects had a negligible influence on the outcomes of this study. Future studies should control for the presence of externalizing problems. Mph-effectiveness on the monitoring of reward and punishment was investigated with a between subjects design with a mixed Mph-free group consisting of Mph-responders off medication as well as a few medication-naïve children. This mixed group limits the generalization of the results to all Mph-free children with ADHD. Despite this limitation, the results are promising in suggesting that Mph significantly alters deviant feedback monitoring in ADHD. Replication with a placebo-controlled within subjects design in a more homogenous ADHD group would allow for more broad conclusions.

Secondly, group differences in task performance might have played a role. First, the task duration was approximately one hour, which might have induced a stronger performance decrement in the children with ADHD (especially without medication) because of sustained attention problems. However, we analysed time-on-task effects, which revealed no group differences in accuracy and faster responses only during the second quarter of an hour in the ADHD groups (see Figure S5 for a description of these results). The absence of a performance decrement in ADHD is in agreement with literature that provides only limited evidence for a stronger performance decrement in ADHD during vigilance tasks [60], [61]. Group differences in performance decrement are therefore unlikely to have influenced the outcomes of the present study. The ADHD groups however made ∼3% more late reactions than the TD group, which resulted in ∼5 cents more punishment in both the reward and punishment blocks for the ADHD groups. This difference also did not moderate the feedback*group effects found in this study (the LPP group differences remained intact when controlling for the percentage of late responses).

Lastly, no statistical correction for multiple comparisons was performed which increases the likelihood of type-I error. With an adjustment of the p-value, important findings would have been lost. In this context, it has to be considered that the significant differences of the study are largely consistent with effect sizes which were of medium to large size. Nevertheless, this is a weakness of the present study.

### Conclusion

Our study provides evidence that *as a group* Mph-free children with ADHD show a relatively intact early attentional selection of feedback (normal feedback P2) but deviant late processing (enhanced feedback LPP) of continuously provided punishment and reward. The latter effect could only partly be explained by comorbid externalizing problems. These findings are in line with ADHD models presuming that the monitoring of external feedback is relatively intact in individuals with ADHD [10], [17] and may explain the often reported behavioural evidence that immediate and/or relatively intense reinforcement has a positive effect on task performance in children with ADHD [8]. The enhanced LPP to the feedback stimuli in our children with ADHD might even explain why the provision of appropriate reinforcement in some studies even has a more positive effect than in control children [8]. Yet, these group results cannot be generalised to all children with ADHD. The correlational findings indicate that children with ADHD-combined type with more inattention problems showed reduced P2 amplitudes to feedback stimuli, but enhanced LPP amplitudes to non-reward and punishment. This suggests that in children with ADHD with more severe inattention problems early attentional selection of feedback stimuli is compromised, while more attention is paid to the affective value of negative feedback stimuli. The Mph-treated children with ADHD did not differ from the TD group, suggesting that Mph normalizes late feedback evaluation processes, however these findings should be replicated in a placebo-controlled within subjects design to draw more firm conclusions about causality.

## Supporting Information

Figure S1 **Topographical maps of the feedback P2 (170–220 ms).** Topographical maps of the feedback P2 in the interval of 170–220 ms after feedback onset on gain, no won, no loss and loss trials, separated for the TD, Mph-treated and Mph-free ADHD groups.
(TIF)

Figure S2 **Topographical maps of the FRN (260–360 ms).** Topographical maps of the feedback related negativity (FRN) in the interval of 260–360 ms after feedback onset on gain, no won, no loss and loss trials, separated for the TD, Mph-treated and Mph-free ADHD groups.
(TIF)

Figure S3 **Topographical maps of the FRN difference potentials (260–360 ms).** Topographical maps of the difference potentials for the feedback related negativity (FRN) in the interval of 260–360 ms after feedback onset for the gain minus no gain comparison and the no loss minus loss comparison, separated for the TD, Mph-treated and Mph-free ADHD groups.
(TIF)

Figure S4 **Topographical maps of the LPP (450–800 ms).** Topographical maps of the LPP in the interval of 450–800 ms after feedback onset on gain, no win, no loss and loss trials, separated for the TD, Mph-treated and Mph-free ADHD groups.
(TIF)

Figure S5 **Time on task effects on accuracy and RT separated for the TD, Mph-treated and Mph-free ADHD groups.** As the task duration was ∼60 minutes, time on task may have had a differential influence on the task performance, and therefore on the brain processes, in the TD and ADHD groups. In order to check whether this may have influenced the outcomes, time on task effects were explored by computing the mean RT and percentage of accurate responses for four quartiles of the task. All quartiles consist of 3 successive task blocks (e.g. quartile 1 consists of the first 3 task blocks, which is ∼15 minutes) (A) Accuracy decreased with time on task (*F*(3,141) = 11.0, p<.001, *η^2^* = .19) from quartile 1 to 2 (p<.001), and with a trend to significance from quartile 2 to 3 (p = .076) and quartile 3 to 4 (p = .056). The groups did not differ from each other in this time on task effect (*F*(6,141) = 0.65, p>.05, *η^2^* = .03), for none of the contrasts. (B) RT decreased with time on task (*F*(3,141) = 16.1, p<.001, *η^2^* = .26), from quartile 1 to 2 (p<.001) and quartile 2 to 3 (p<.01). Although the groups did not differ in the overall time on task effect for RT (*F*(6,141) = 1.2, p>.05, *η^2^* = .05), contrasts between the quartiles revealed that the groups differed in the reduction of RT from quartile 1 to 2 (*F*(1,47) = 6.2, p<.01, *η^2^* = .21). Post hoc analyses between the groups indicated that both ADHD groups showed a reduction in RT for this contrast whereas the TD group did not.
(TIF)
