# Supplementary figures and images for: Processing of Continuously Provided Punishment and Reward in Children with ADHD and the Modulating Effects of Stimulant Medication: An ERP Study
Source: PLoS One. 2013 Mar 21;8(3):e59240. doi: 10.1371/journal.pone.0059240 (PMC3605450; doi:10.1371/journal.pone.0059240)

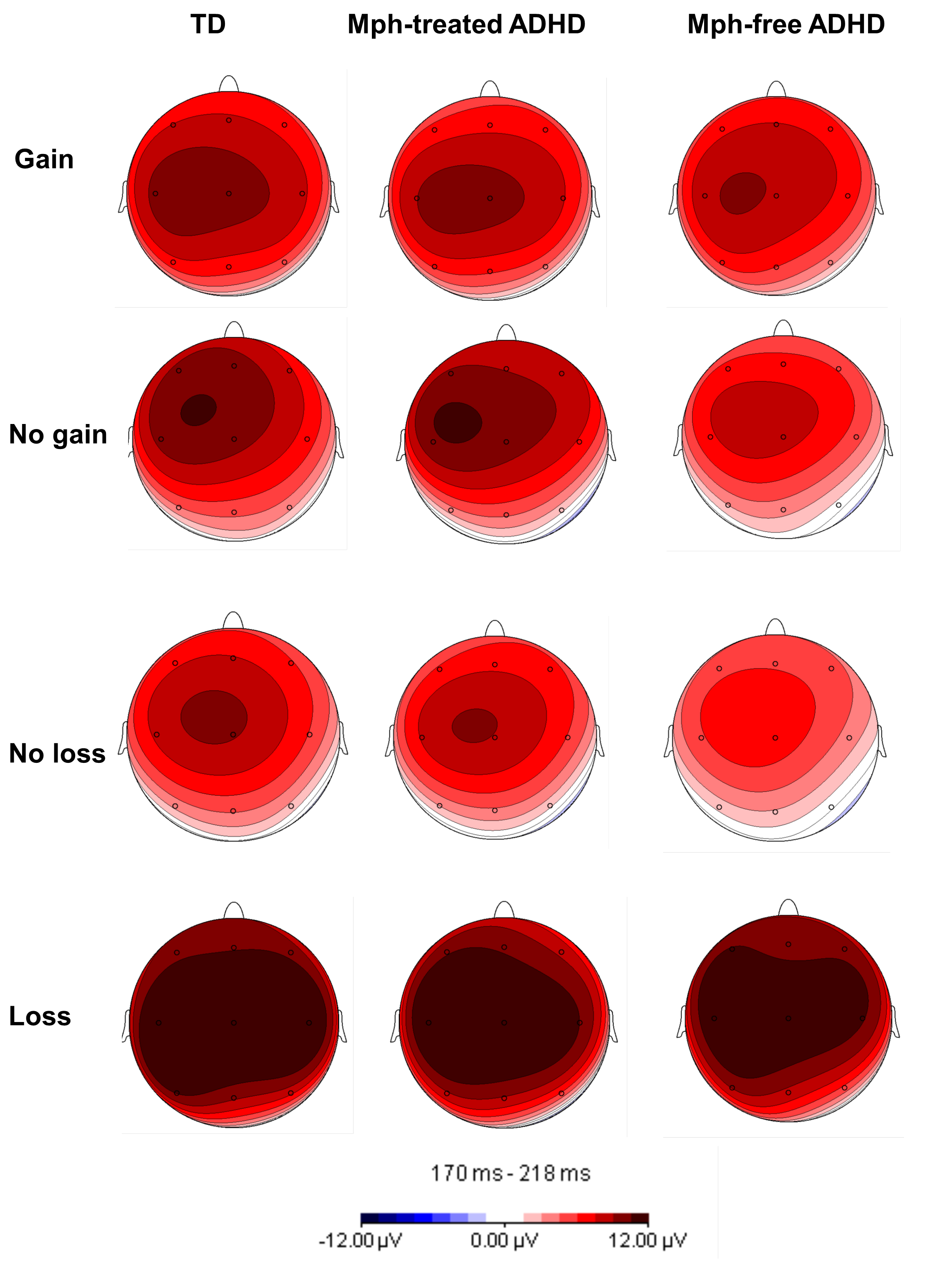

Supplement: Figure S1 — Topographical maps of the feedback P2 (170–220 ms). Topographical maps of the feedback P2 in the interval of 170–220 ms after feedback onset on gain, no won, no loss and loss trials, separated for the TD, Mph-treated and Mph-free ADHD groups. (TIF) [file pone.0059240.s001.tif]

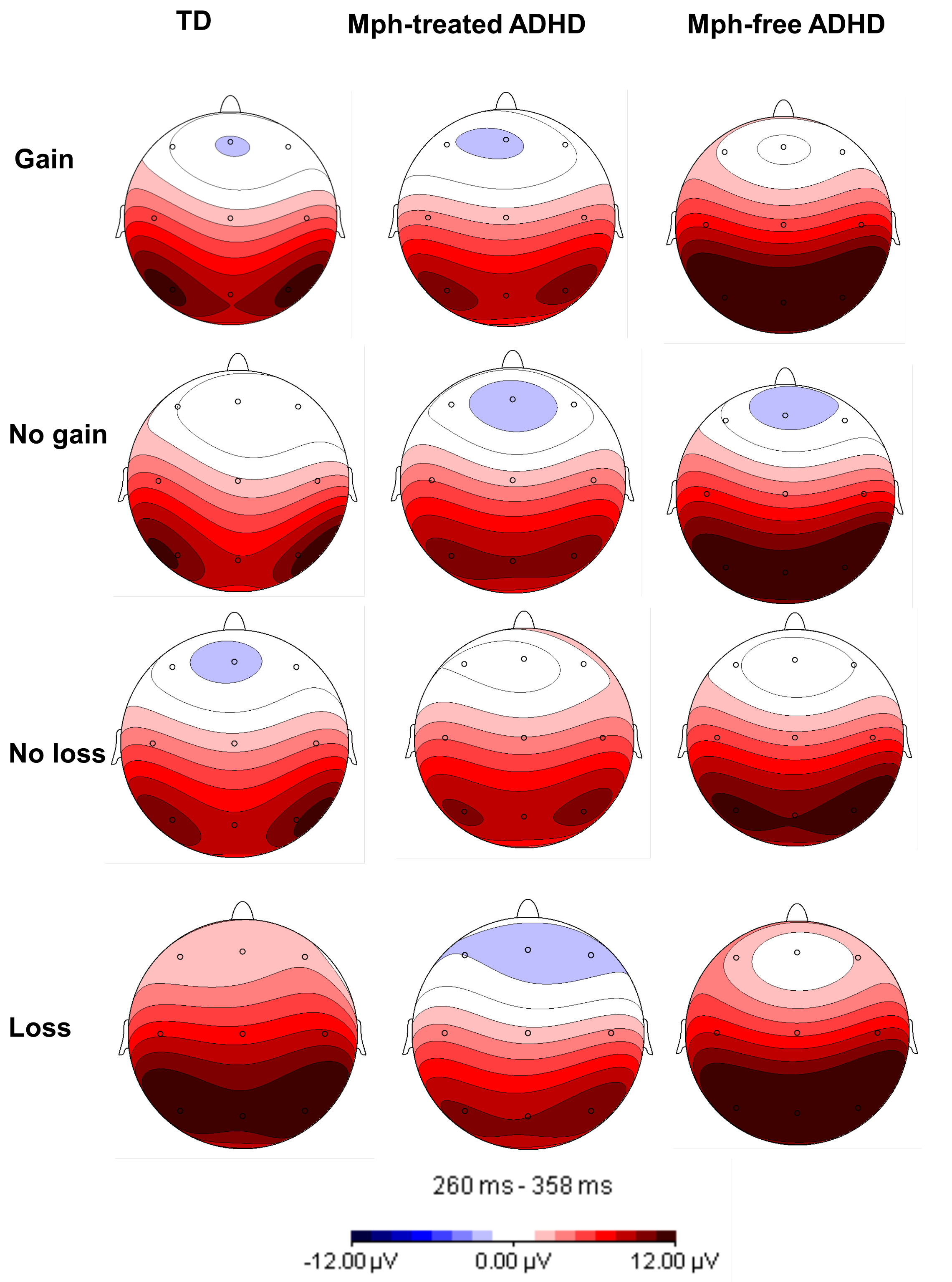

Supplement: Figure S2 — Topographical maps of the FRN (260–360 ms). Topographical maps of the feedback related negativity (FRN) in the interval of 260–360 ms after feedback onset on gain, no won, no loss and loss trials, separated for the TD, Mph-treated and Mph-free ADHD groups. (TIF) [file pone.0059240.s002.tif]

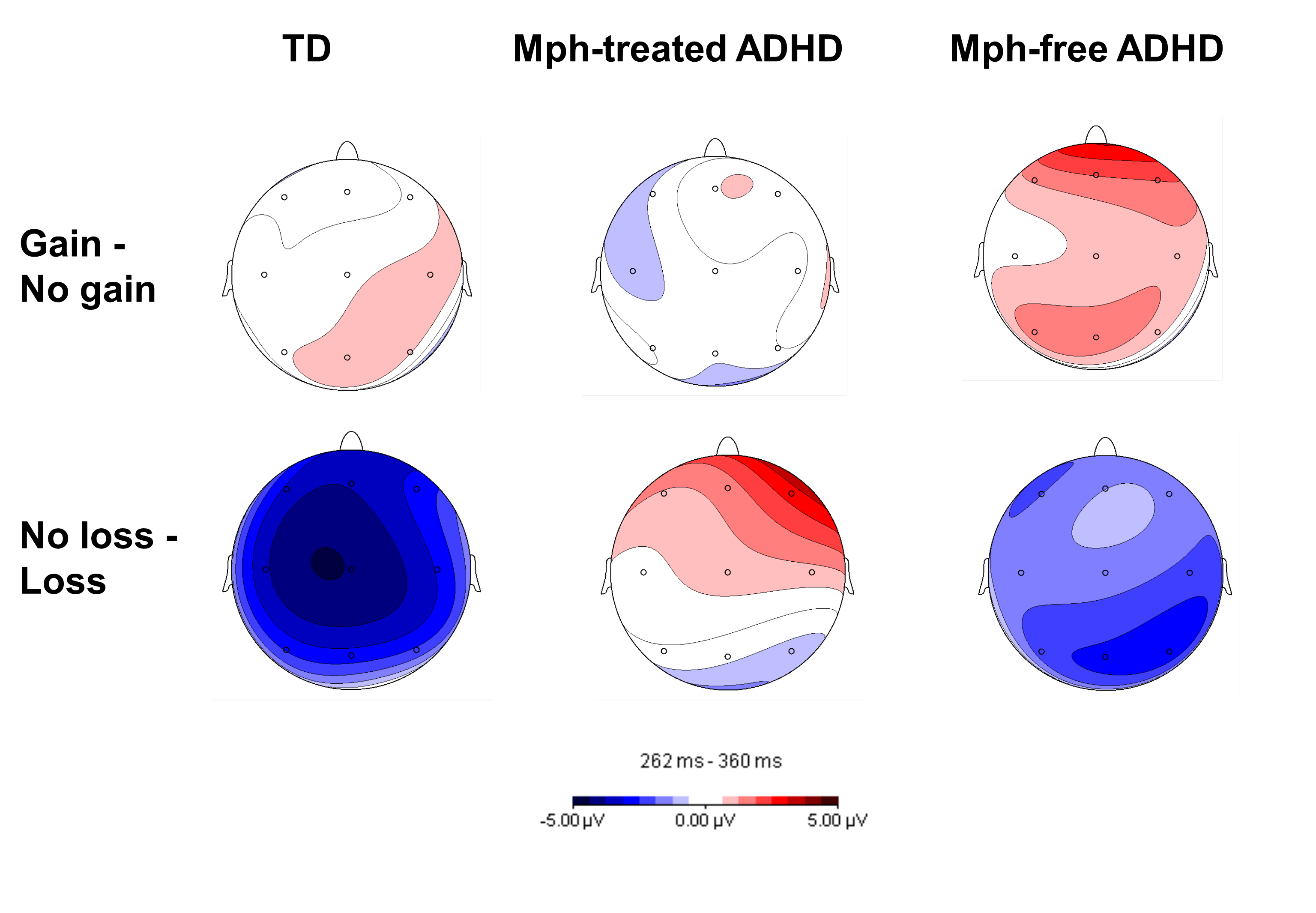

Supplement: Figure S3 — Topographical maps of the FRN difference potentials (260–360 ms). Topographical maps of the difference potentials for the feedback related negativity (FRN) in the interval of 260–360 ms after feedback onset for the gain minus no gain comparison and the no loss minus loss comparison, separated for the TD, Mph-treated and Mph-free ADHD groups. (TIF) [file pone.0059240.s003.tif]

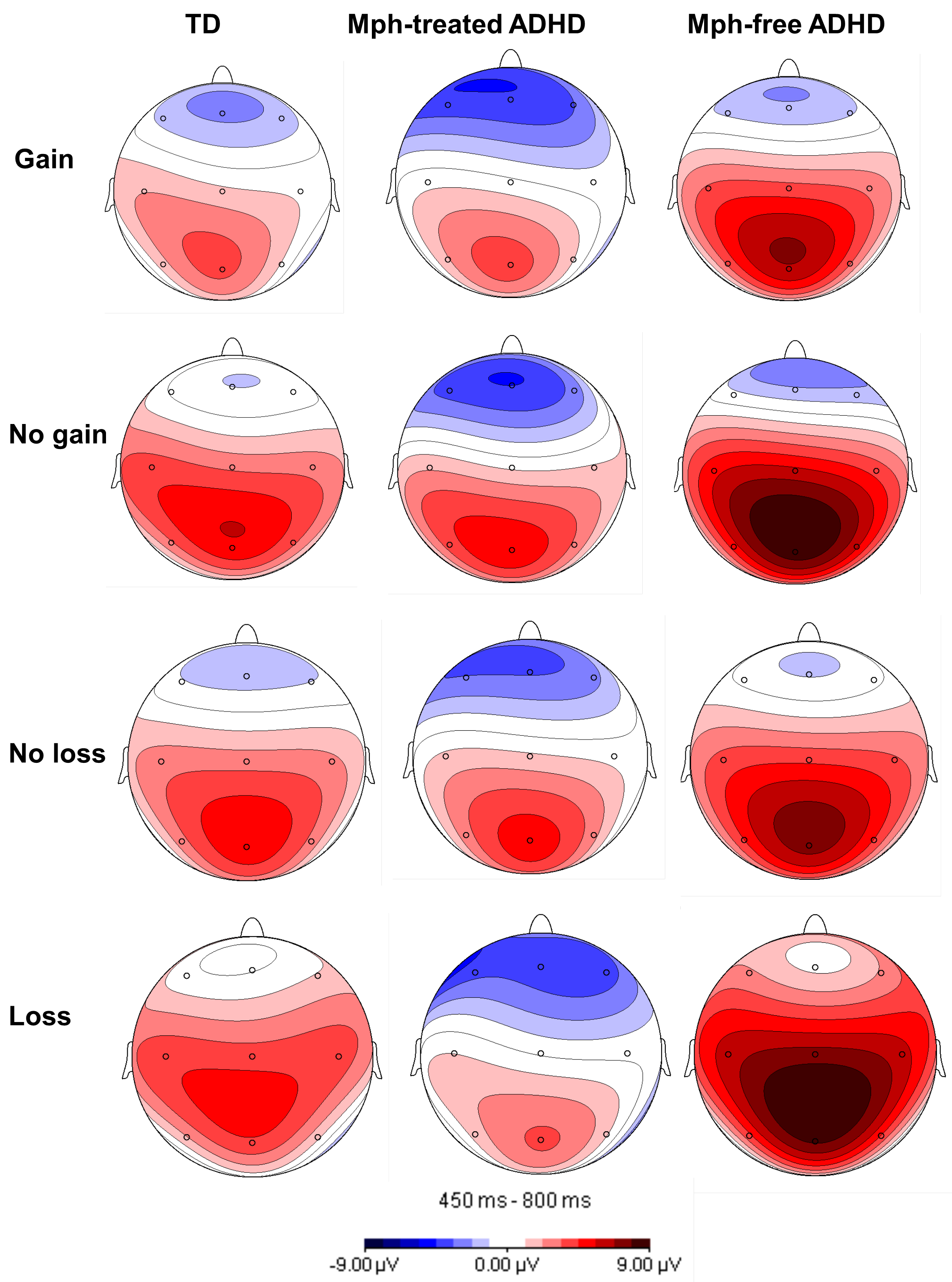

Supplement: Figure S4 — Topographical maps of the LPP (450–800 ms). Topographical maps of the LPP in the interval of 450–800 ms after feedback onset on gain, no win, no loss and loss trials, separated for the TD, Mph-treated and Mph-free ADHD groups. (TIF) [file pone.0059240.s004.tif]
